# Mind the gap! transition from child & adolescent to adult mental health services: A narrative review and results of 18 months consultation

**DOI:** 10.1192/j.eurpsy.2021.1072

**Published:** 2021-08-13

**Authors:** R. Cajão, M. Martins, J. Estrada, G. Lima

**Affiliations:** Psychiatry, Centro Hospitalar Barreiro-Montijo, Barreiro, Portugal

**Keywords:** transition, CAMHS, AMHS, adolescent

## Abstract

**Introduction:**

Discontinuity in child and adolescent mental health services (CAMHS) and adult mental health services (AMHS) constitutes an important challenge in mental health care. In the last decade, efforts have been made to better define the transitioning population and build consensual models for CAMHS-AMHS’ transition.

**Objectives:**

We aim to present our protocol and transition consultation results on the scope of published literature.

**Methods:**

Description of protocol and casuistic of 18 months’ transition consultation at Centro Hospitalar Barreiro-Montijo. The literature found on PubMed was published from 2008 to 2020 and was reviewed using the keywords: transition, CAMHS, AMHS, adolescent, mental health service, young people. Articles with full text available written in English and French were selected. The included clinical studies focused on populations with neurodevelopmental disorders, psychotic disorders, non-suicidal self-harm and suicidal attempts.

**Results:**

Forty-four articles were included, published from 2008 to 2020. 4 articles were excluded on basis of language and diagnosis criteria (eating disorders). Twelve were reviews, 24 were clinical studies and 4 were opinion articles. There are cultural and referral issues that explain the loss of patients in this transition gap. Individuals with history of severe mental illness were more frequently referred than those with neurodevelopmental disorders. Optimal transition is defined as adequate transition planning with a flexible age cut-off and continuity of care following transition.

**Conclusions:**

For the vast majority of service users, transition from CAMHS to AMHS is poorly planned, executed and experienced. Improving transition models is essential to the patients autonomy’ promotion and a stronger adult mental health.

